# Latent class analysis of imaging and clinical respiratory parameters from patients with COVID-19-related ARDS identifies recruitment subphenotypes

**DOI:** 10.1186/s13054-022-04251-2

**Published:** 2022-11-25

**Authors:** Daan F. L. Filippini, Elisa Di Gennaro, Rombout B. E. van Amstel, Ludo F. M. Beenen, Salvatore Grasso, Luigi Pisani, Lieuwe D. J. Bos, Marry R. Smit

**Affiliations:** 1 Department of Intensive Care Medicine, Amsterdam UMC, University of Amsterdam, 1105AZ Amsterdam, The Netherlands; 2 Faculty of Medicine, University of Bari, Bari, Italy; 3 Department of Radiology and Nuclear Medicine, Amsterdam UMC, University of Amsterdam, Amsterdam, The Netherlands; 4 Department of Emergency and Organ Transplantation, University of Bari Aldo Moro, Bari, Italy; 5 Critical Care Africa Asia Network, Mahidol Oxford Research Unit, Bangkok, Thailand; 6 Department of Anesthesia and Intensive Care, Miulli Regional Hospital, Acquiviva Delle Fonti, Bari, Italy; 7 Department of Pulmonology, Amsterdam UMC, University of Amsterdam, Amsterdam, The Netherlands; 8 Laboratory of Experimental Intensive Care and Anesthesiology (L.E.I.C.A.), University of Amsterdam, Amsterdam, The Netherlands

**Keywords:** COVID-19, ARDS, Latent class analysis, Phenotypes, Recruitment, Respiratory parameters, Radiological data, Mechanical ventilation

## Abstract

**Background:**

Patients with COVID-19-related acute respiratory distress syndrome (ARDS) require respiratory support with invasive mechanical ventilation and show varying responses to recruitment manoeuvres. In patients with ARDS not related to COVID-19, two pulmonary subphenotypes that differed in recruitability were identified using latent class analysis (LCA) of imaging and clinical respiratory parameters. We aimed to evaluate if similar subphenotypes are present in patients with COVID-19-related ARDS.

**Methods:**

This is the retrospective analysis of mechanically ventilated patients with COVID-19-related ARDS who underwent CT scans at positive end-expiratory pressure of 10 cmH_2_O and after a recruitment manoeuvre at 20 cmH_2_O. LCA was applied to quantitative CT-derived parameters, clinical respiratory parameters, blood gas analysis and routine laboratory values before recruitment to identify subphenotypes.

**Results:**

99 patients were included. Using 12 variables, a two-class LCA model was identified as best fitting. Subphenotype 2 (*recruitable*) was characterized by a lower PaO_2_/FiO_2_, lower normally aerated lung volume and lower compliance as opposed to a higher non-aerated lung mass and higher mechanical power when compared to subphenotype 1 (*non-recruitable*). Patients with subphenotype 2 had more decrease in non-aerated lung mass in response to a standardized recruitment manoeuvre (*p* = 0.024) and were mechanically ventilated longer until successful extubation (adjusted SHR 0.46, 95% CI 0.23–0.91, *p* = 0.026), while no difference in survival was found (*p* = 0.814).

**Conclusions:**

A *recruitable* and *non-recruitable* subphenotype were identified in patients with COVID-19-related ARDS. These findings are in line with previous studies in non-COVID-19-related ARDS and suggest that a combination of imaging and clinical respiratory parameters could facilitate the identification of recruitable lungs before the manoeuvre.

**Supplementary Information:**

The online version contains supplementary material available at 10.1186/s13054-022-04251-2.

## Background

Patients with novel coronavirus disease 2019 (COVID-19) frequently develop acute respiratory distress syndrome (ARDS) and require invasive mechanical ventilation [1]. ARDS has a high mortality and is characterized by acute diffuse inflammatory lung injury, leading to increased pulmonary vascular permeability, increased lung weight and loss of aerated lung tissue [2, 3]. However, not all patients have similar injury mechanisms and patterns, resulting in biological and physiological heterogeneity, possibly explaining the lack of effective treatments in unselected ARDS patients [4–7]. A better understanding of ARDS heterogeneity can help to provide more targeted treatments, for example, by only providing a higher positive end-expiratory pressure (PEEP) strategy to patients with recruitable lung tissue [8, 9].

Patients with COVID-19-related ARDS (COVID-ARDS) show substantial heterogeneity in response to recruitment manoeuvres, but this difference can only be observed after the manoeuvre has been performed [10]. In patients with ARDS not related to COVID, two subphenotypes were identified using latent class analysis (LCA) of data on computed tomography (CT) measures of lung tissue, respiratory parameters and gas exchange measures [11]. These subphenotypes responded differently to recruitment manoeuvres and might therefore require another PEEP strategy. The *recruitable* subphenotype demonstrated a lower respiratory system compliance and PaO_2_/FiO_2_, a higher fraction of dead space and a more inhomogeneous lung parenchyma injury compared to the *non-recruitable subphenotype*. However, it remains unclear whether the *recruitable* and *non-recruitable* subphenotypes established in non-COVID-19-related ARDS (non-COVID-ARDS) can be extended to COVID-ARDS.

The aim of our study is to identify respiratory subphenotypes within COVID-ARDS by using LCA on respiratory parameters, gas and tissue volumes derived from CT, blood gas analysis and routine laboratory results. We hypothesize that the *recruitable* and *non-recruitable* subphenotypes within non-COVID-ARDS are also observable in patients with COVID-ARDS.

## Methods

### Study design, patients and ethics

A retrospective cohort study was conducted of patients admitted to the intensive care unit (ICU) of a large university hospital; the Amsterdam University Medical Centres, location AMC between April 2020 and April 2021. We analysed all patients who (1) were intubated for COVID-ARDS, defined according to the Berlin criteria [2], and (2) received CT scans at 10 cmH_2_O and 20 cmH_2_O PEEP, with a recruitment manoeuvre between scans. Per hospital protocol, each patient with COVID-ARDS who underwent a CT scan was imaged at those two PEEP levels with a recruitment manoeuvre in between. These recruitment manoeuvres were performed as part of standard practice to inform the physician about the recruitability of consolidated lung tissue. The institutional review board approved the study protocol and waived the need of informed consent.

### CT scan and data collection

Non-enhanced chest CT scans were acquired at 10 cmH_2_O PEEP (PEEP before recruitment) and after a recruitment manoeuvre at a PEEP level of 20 cmH_2_O (PEEP after recruitment). Both scans were acquired in the end-expiratory phase. In-between the two CT scans, a Hamilton C2 ventilator was used to deliver 3 sustained inflations lasting 10 s by an inspiratory hold, increasing the airway pressure to 40 cm H_2_O for the entire hold.

Shortly before the CT scans, clinically available respiratory parameters and blood gas results were collected. Formulas used to calculate additional respiratory parameters and more details on data collection are listed in the supplementary materials. Besides that, routine laboratory results and patient demographics were collected.

### CT Quantitative analysis

Lung tissue in the CT scans was segmented by an open-source artificial intelligence algorithm [12] and then manually adjusted using ITK-Snap [13]. Segmentation consisted of drawing the outline of the lungs in each CT slice, excluding hilar vessels, the main bronchi, and if present pleural effusions, pneumothorax and pneumomediastinum areas. The segmentation method depended on the available slice thickness: for the patients with a CT scan composed of 3-mm slices, all slices were segmented. For patients with a CT scan consisting of 1-mm slices, a reduced number of slices were extrapolated and segmented in order to make the quantitative analysis more efficient. This was done similar to previous studies that validated the use of a reduced amount of slices for an accurate evaluation of lung aeration [14, 15]. The distance between the 1-mm slices was set at 20 mm, as previously suggested [16].

The determination of gas and tissue volumes was performed by analysing CT numbers of all lung voxels in Hounsfield units (HU). Lung regions were classified as normally aerated (from − 900 to − 501 HU), poorly aerated (from − 500 to − 101 HU), non-aerated (from − 100 to 100 HU) and hyper-inflated (from − 1000 to − 901 HU) to allow for comparison with previous ARDS literature [3].

To calculate lung volume, the number of lung voxels was multiplied by the volume of one voxel in millilitres to form the total volume of the lung irrespective of aeration of the tissue. As lung tissue is assumed to be a composition of air (− 1000 HU) and lung parenchyma with a similar density to water (0 HU), lung weight could be calculated using the tissue fraction of the lung derived from CT numbers [16, 17]. End-expiratory lung volume was calculated using the gas fraction of the lung. Recruitment was defined as the decrease in non-aerated lung weight after the recruitment manoeuvre, divided by total lung weight before the recruitment manoeuvre.

### Subphenotype identification

LCA was applied on clinically available respiratory parameters, blood gas analysis, CT-derived gas and tissue volumes (at PEEP before recruitment) and routine laboratory results. Outcomes and demographics were not considered during model design and LCA.

Variables that correlated considerably (Spearman correlation coefficient > 0.7 and *p* value < 0.05) or were mathematically coupled were excluded from the LCA, as variables are assumed to be locally independent and a violation of that assumption could introduce bias [18, 19]. Data used in the LCA were imputed (supplementary materials) and transformed to resemble a normal distribution, which was verified using histograms, Q–Q plots and the Shapiro–Wilk test. Variables were scaled by subtracting the mean and dividing by the standard deviation. The best fitting latent model in terms of number of latent classes was evaluated by the Bayesian information criterion (BIC) score and Lo–Mendell–Rubin adjusted likelihood ratio test (LMR-LRT) [20]. Entropy, median class assignment probabilities and the amount of class assignment probabilities above 90 per cent were calculated. During model design and LCA, key steps and considerations described by Sinha et al. [19] were taken into account. Subphenotype characteristics were displayed using a profile plot containing standardized mean differences (SMDs) of subphenotype defining variables. To perform the LCA, open-source package ‘Flexmix’ was used with model driver ‘FLXMCmvcombi’ which allows for both binary and Gaussian indicators as input.

### Statistical analysis

Continuous data were expressed as mean with the standard deviation or median with the interquartile range according to statistical distribution. Categorical data were presented as numbers with percentages. Demographical parameters, clinical parameters, CT-derived lung volumes and weight and outcomes were compared between subphenotypes. Differences in mean, median and proportion between subphenotypes were tested using t-test, Wilcoxon signed-rank test or chi-squared test as appropriate. Tests were two-sided with a type I error set at 5%.

For a simplified subphenotype identification, a nested parsimonious model was created considering all LCA variables and tested for subphenotype prediction capacity. To create the nested model, all variables were entered in a LASSO regression, while tuning its *λ* parameter in order to arrive at a 4 variable model. Next, these variables were entered in a generalized linear model (GLM) validated with fivefold cross-validation. Finally, to quantify, assess and compare the subphenotype prediction performance of the nested models, areas under the receiver operating characteristics curve (AUROC) and bootstrapped confidence intervals were calculated [21]. An additional nested parsimonious model excluding CT-derived parameters was also created and tested, as well as standard ICU severity scores PaO_2_/FiO_2_, Apache II and SOFA.

Survival was visualized using Kaplan–Meier curves and analysed using a Cox proportional hazards model. Time until successful extubation and survival was analysed in a Fine and Gray competing risks regression model and displayed in a cumulative incidence function plot. The subdistribution hazard ratio (SHR) was calculated representing time until successful extubation in the presence of diverging survival. Both the survival and the competing risk analysis were corrected for predefined confounders: age, gender and Apache II [22] score. Survival and time until successful extubation were treated as right-censored data, with censoring representing having left the ICU alive or completing the 60-day follow-up period.

All analyses were performed with *R* through the *R*-studio interface, version 4.0.3.

## Results

### Population

**Fig. 1:**
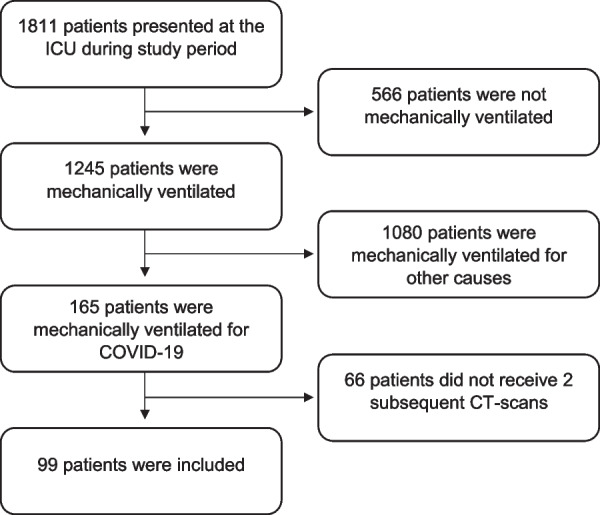
Flowchart of the inclusion process

A total of 99 mechanically ventilated COVID-ARDS patients were included in the analysis (Fig. 1). Baseline characteristics are presented in Table 1. The mean age was 63 years (SD ± 10), and most patients were male (68.7%). The median time between ICU admission and CT scans was 5 days [IQR: 1–11] and 43 out of 99 patients died in the ICU (50.6%).

**Table 1:** Baseline characteristics

	All patients	Subphenotype 1 (non-recruitable)	Subphenotype 2 (recruitable)	*p* value
n	99	62	37	
*Demographics*				
Age (years)	63 (10)	65 (9)	60 (10)	0.008
Gender = Male (%)	68 (69)	45 (73)	23 (62)	0.391
BMI (kg m^−2^)	29.18 (6.29)	28.57 (5.37)	30.19 (7.55)	0.218
COVID symptoms duration (days)	9 [5, 12]	8 [5, 10]	10 [7, 14]	0.027
COVID ICU stay at inclusion (days)	5 [1, 11]	5 [1, 9]	7 [0, 12]	0.633
*Respiratory parameters*				
Respiratory rate (min^−1^)	25 (7)	24 (7)	28 (7)	0.003
Tidal volume (mL)	444 (131)	440 (123)	451 (144)	0.681
Tidal volume / IBW (mL kg^−1^)	6.47 [5.67, 7.90]	6.36 [5.69, 7.63]	6.76 [5.67, 7.90]	0.395
Arterial pH	7.37 [7.30, 7.43]	7.38 [7.34, 7.44]	7.32 [7.26, 7.42]	0.005
PEEP before recruitment (cmH_2_O)	10 [8, 12]	10 [8, 11]	10 [10, 12]	0.008
Driving pressure (cmH_2_O)	12 [8, 17]	10 [7, 13]	15 [12, 20]	< 0.001
Ventilatory ratio	1.57 [1.23, 2.40]	1.43 [1.12, 1.92]	2.40 [1.38, 3.05]	0.001
Compliance of respiratory system (cmH_2_O)	33 [24, 58]	42 [28, 73]	29 [21, 39]	0.004
*Severity*				
Apache II score	15 [11.5, 20.5]	20 [12, 21.75]	12 [10, 20]	0.042
PaO_2_/FiO_2_ (mmHg)	106 [80, 138]	130 [98, 158]	81 [72, 103]	< 0.001
ARDS category (%)				< 0.001
Mild	6 (6.2)	6 (10)	0 (0)	
Moderate	47 (48.5)	36 (60)	11 (29.7)	
Severe	44 (45.4)	18 (30)	26 (70.3)	
*Outcomes*				
Duration of mechanical ventilation (days)	16 [9, 27]	15 [7.5, 25.5]	16 [12, 27]	0.189
Duration of ICU stay (days)	18 [11, 31]	18 [10, 30.5]	17.5 [13, 32.5]	0.266
Successfully extubated (%)	39 (46)	27 (53)	12 (35)	0.168
ICU mortality (%)	43 (51)	23 (45)	20 (59)	0.308

Data are shown for the entire cohort and stratified for the two subphenotypes identified by the latent class analysis. *BMI* Body mass index, *COVID-19* coronavirus disease 2019, *ICU* intensive care unit, *IBW* ideal body weight, *PEEP* positive end-expiratory pressure

### Latent class analysis

**Fig. 2:**
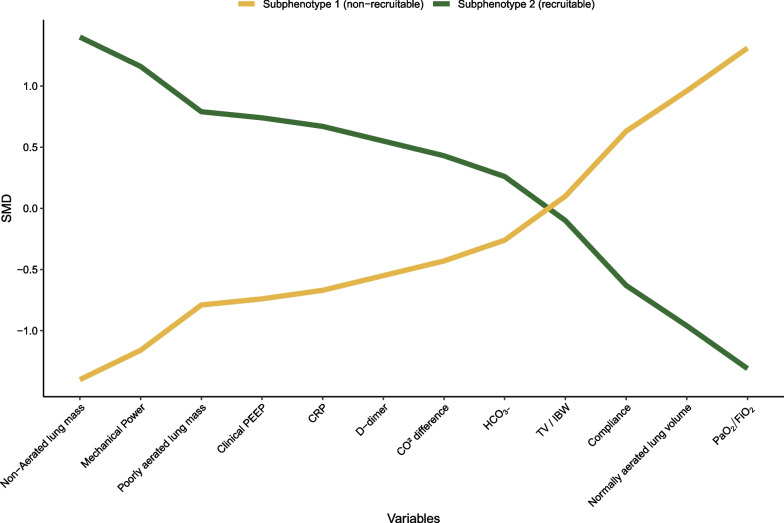
Profile plot of the two subphenotypes identified by the LCA. All variables used in the latent class analysis are plotted on the *x*-axis, with the *y*-axis displaying the standardized mean difference (SMD) of the corresponding variables in both of the LCA derived subphenotypes. SMDs are calculated by standardizing the variable to a mean of 0 and a standard deviation of 1. The variables on the *x*-axis are ordered by the *y*-value of the recruitable subphenotype in a descending way. *TV* Tidal volume, *IBW* ideal body weight, *PEEP* positive end-expiratory pressure

A total of 25 variables were considered for the LCA. After discarding correlated and mathematically coupled variables, 12 variables were used in the LCA (Fig. 2 and Additional file 1: Fig. S1). Table 2 shows model-fitting statistics for LCA models consisting of one to five classes. The LMR-LRT, which tests if a model with *n* classes provides an improved fit compared to a model with *n-1* classes, showed a p value lower than 0.001 for all numbers of classes. BIC was lowest for a two-class model, implying that another number of classes would not increase the distinction of the classes without also overfitting them. Because of these findings and small size of the subsets in a three-class solution, a two-class latent model was judged as most suitable.

**Table 2:** Model-fit statistics for different numbers of latent classes

Classes	No. of patients per class	BIC	LMR-LRT	LMR-LRT *p* value	Entropy
1	99	3469.681	–	–	–
2	62, 37	3444.222	130.8	< 0.001	0.75
3	53, 19, 27	3454.702	97.3	< 0.001	0.88
4	18, 43, 35, 13	3484.582	79.2	< 0.001	0.91
5	36, 22, 13, 14, 14	3539.557	55.9	< 0.001	0.93

*BIC* Bayesian information criterion. *LMR-LRT* Lo–Mendell–Rubin adjusted likelihood ratio test

1.3% of LCA variables were missing. The mean imputation effect on subphenotype identification (i.e. patients that switched between classes) was 3%, while outcomes and subphenotype characteristics were comparable between the imputation models (Additional file 1: Table S1, Fig. S2). Between the complete case analysis and the first imputation model, a subphenotype misclassification of 7 (8%) was found, with comparable subphenotype characteristics, outcomes and recruitability (Additional file 1: Table S1, Figs. S3 and S4). Therefore, the results used in the article were based on the first imputation set while additionally validating all outcomes using the complete case analysis.

The two-class latent model assigned 62 (62.6%) patients to subphenotype 1 and 37 (37.4%) to subphenotype 2. Entropy, a measure indicating class distinction without correcting for overfitting, of 0.75 was accepted in light of the number of variables used in the LCA and the sample size. The median class assignment probability was 98.5% [IQR: 89.7–100] for subphenotype 1 and 99.6% [IQR: 95.4–100%] for subphenotype 2. The number of patients with a class assignment probability above 90% was 46 (74%) in subphenotype 1 and 29 (78%) in subphenotype 2.

### Subphenotype characteristics and identification

**Fig. 3:**
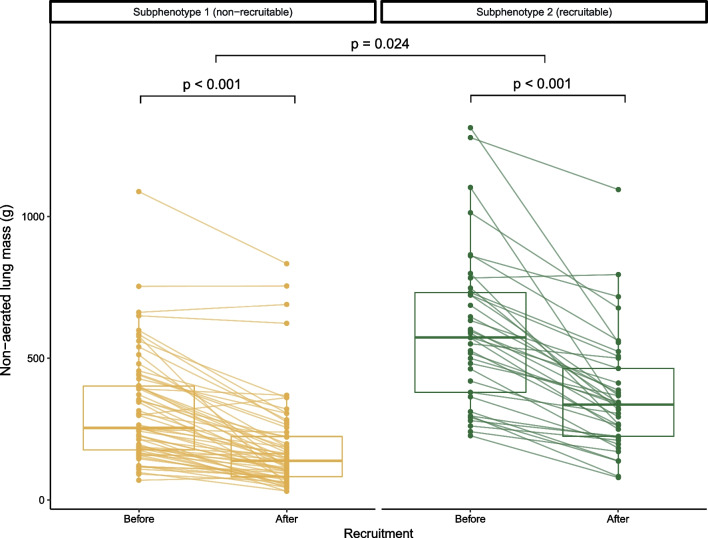
Non-aerated lung mass before and after the recruitment manoeuvre. Data are stratified and coloured by subphenotype. The *y*-axis shows non-aerated lung mass in grams, derived by quantitative CT analysis. Corresponding patient data points are connected by a line. The p values before and after recruitment (bottom *p* values) compare relative amounts (grams/total grams) by the recruitment manoeuvre and are derived by Wilcoxon signed-rank tests. The *p* value between subphenotypes (upper *p* value) compares the changes in relative amounts by the recruitment manoeuvre (change in grams/total grams) between subphenotypes and is derived by a Mann–Whitney *U* test (Additional file 1: Table S2)

Subphenotype 2 had a higher non-aerated lung mass and a higher mechanical power as opposed to a lower normally aerated lung volume, a lower compliance and lower PaO_2_/FiO_2_ when compared to subphenotype 1 (Fig. 2, Table 1 and Additional file 1: Table S2). Subphenotype 2 showed a higher percentage of recruitable lung when compared to subphenotype 1; 12.56% [IQR: 6.72, 18.17] versus 8.95% [IQR: 3.68, 14.25], respectively (*p* = 0.024, Figs. 2 and 3, Additional file 1: Tables S1, S2). Because of these findings, we further refer to subphenotype 2 as *recruitable* and subphenotype 1 as *non-recruitable*. CT-derived lung volumes and weights before and after the recruitment manoeuvre are displayed in the supplementary materials (Additional file 1: Table S2, Figs. S5 and S6).

In terms of simplified subphenotype prediction, the LASSO regression arrived at a four variable nested model consisting of PaO_2_/FiO_2_, normally aerated lung volume, non-aerated lung mass and mechanical power (Additional file 1: Tables S3 and S4). This model had excellent diagnostic accuracy for subphenotype identification, with an AUROC of 0.93 (95% CI 0.88–0.98, Additional file 1: Table S5, Fig. S7) [21]. The predictive capacity of the additional subphenotype prediction model excluding CT-derived parameters was also excellent (AUROC 0.87, 95% CI 0.79–0.91), while separate ICU severity scores showed poor to good AUROCs: SOFA score 0.51 [95% CI 0.34–0.58], Apache II score 0.62 [95% CI 0.48–0.72] and PaO_2_/FiO_2_ 0.79 [95% CI 0.48–0.72] (Additional file 1: Tables S3–S5, Fig. S7).

### Outcome differences between subphenotypes

**Fig. 4:**
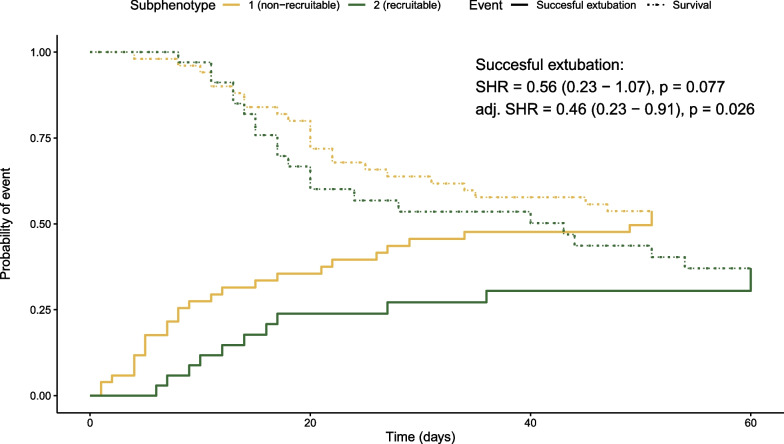
Cumulative incidence function curves of ‘survival’ and ‘time until successful extubation’. Data are stratified and coloured by subphenotype. Time in days is displayed on the *x*-axis and the probability of an event on the *y*-axis. The annotated subdistribution hazard ratios (SHRs) compare successful extubation between subphenotypes in the presence of survival and are derived by means of a Fine and Gray competing risk analysis. Crude SHR and adjusted (age, gender and Apache II) SHR are presented, with the 95% confidence interval displayed between parentheses

ICU mortality, ICU length of stay, duration of mechanical ventilation (MV) and successful extubation rate were individually not different between subphenotypes (Additional file 1: Fig. S1). In a survival analysis, no difference was found between subphenotypes (HR = 1.08, 95% CI 0.58–1.98, *p* = 0.814, Additional file 1: Tables S1, S6 and S7, Figs. S8 and S9) and adjusting for confounders (age, gender, Apache II) did not alter those results. When analysing duration of MV until successful extubation with mortality as a competing risk, the non-recruitable subphenotype showed a reduced duration of MV until successful extubation after correcting confounders (adjusted SHR = 0.46, 95% CI 0.23–0.91, p = 0.026, Fig. 4, Additional file 1: Table S6). Outcomes were similar in the complete case analysis (Additional file 1: Table S8, Figs. S10 and S11).

## Discussion

In this study, we showed that mechanically ventilated COVID-ARDS patients can be divided into two distinct subphenotypes based on respiratory parameters, blood gas analysis, CT measurements and routine laboratory results. The two subphenotypes have a different response to a standardized recruitment manoeuvre, identifying them as recruitable and non-recruitable. Recruitable subphenotype patients had a longer duration of mechanical ventilation until successful extubation, while no difference between the subphenotypes was found in terms of survival. The recruitable subphenotype was characterized by a lower PaO_2_/FiO_2_ ratio, lower normally lung volume on CT scans, lower compliance, but higher mechanical power and higher mass of non- and poorly aerated lung tissue on CT scans.

The subphenotype characteristics in COVID-ARDS patients are in line with the recruitable and non-recruitable subphenotypes in non-COVID-ARDS patients [11]. Only minor differences were found between the two studies, with the most prominent being the increased significance of mechanical power in the non-recruitable COVID-ARDS subphenotype [11]. The similar characteristics between non-COVID-ARDS and present study are welcome as it improves the external validity of the already existing subphenotypes and implies resemblance between non-COVID-ARDS and COVID-ARDS. The subphenotype characteristics of present study are also consistent with the H-type and L-type COVID-ARDS phenotype hypothesis, which suggest an association between high lung weight on CT, high lung elastance (i.e. low compliance) and high recruitability [23]. We here present a data-driven, multi-dimensional identification of recruitment subphenotypes not solely dependent on lung weight and compliance as parameters to define recruitability.

We did not observe a difference in mortality between patients with a recruitable and non-recruitable subphenotype in patients with COVID-ARDS, which is in contrast to previous findings in non-COVID-ARDS [11]. The direction of effect, with a higher mortality rate in patients with a recruitable subphenotype, however, was consistent between the studies and the lack of significance may be contributed to the sample size. Indeed, the likelihood of successful extubation was lower in patients with the recruitable subphenotype in the present study, an outcome that was not studied in non-COVID-ARDS. Taken together, the aggregated data suggest that recruitable subphenotype is more severely ill, and that the lower likelihood of successful extubation is independent of several important confounders.

To independently reproduce and extend the non-COVID-ARDS subphenotypes to COVID-ARDS, present study conducted an LCA instead of solely using proposed non-COVID-ARDS prediction models [19]. Nonetheless, there are some important methodological differences between present study and the previously mentioned non-COVID-ARDS study. First, the present study assesses recruitment between PEEP levels of 10 cmH_2_O and 20 cmH_2_O, while the non-COVID-ARDS cohort assesses it between PEEP 5cmH_2_O and plateau pressure of 45 cmH_2_O, possibly explaining the lower recruitability percentage found in present study. Second, this study uses only respiratory parameters before recruitment and can therefore not confirm the large improvement of oxygenation and compliance that was found in non-COVID-ARDS. Finally, in non-COVID-ARDS patients dead space has shown to be an important subphenotype characteristic, but that is only directly estimated with volumetric capnography of which measurements were not available in our study. However, ventilatory ratio has shown to be a surrogate for dead space and was included in our study [24]. Consistent with non-COVID-ARDS, it was higher in the recruitable subphenotype.

Contrary to our findings, a previous large COVID-ARDS study did not find respiratory subphenotypes in the first 4 days of mechanical ventilation when using a LCA comparable to present study [25]. This is most likely explained by the different variables used in the LCA (e.g. present study used CT-derived parameters and routine laboratory results) or our median ventilation duration of 5 days at inclusion. However, the previously mentioned study used a longitudinal LCA (an analysis not used in this study) and found two longitudinal subphenotypes. Remarkably, the main discriminatory variables of those longitudinal subphenotypes (mechanical power, minute ventilation, ventilatory ratio) overlap with present study’s subphenotype defining characteristics.

The main strengths of our study include the usability of the results during routine practice, as only clinically available data before recruitment were used to create the subphenotypes on a homogeneous and easily recognizable ARDS cohort thanks to a single detectable underlying cause. Besides that, CT scans were analysed in a quantitative manner that has high validity and reliability and is therefore considered the gold standard for assessing aeration of lung tissue [26]. The present study also knows several limitations. Selection bias might have occurred as the decision to perform a CT scan was not predefined but at the discretion of the treating physician. Another plausible risk for selection bias was the single-centre retrospective study design in a large university hospital. Because of the COVID-19 pandemic, however, COVID-ARDS patients were redistributed evenly throughout the country minimizing this bias. Considering the LCA, the sample size was relatively small, increasing the possible influence of missing variables. Multiple imputation sets were included in the study which demonstrated a minimal effect on subphenotype identification. Finally, some patients were ventilated using pressure support mode which limits the interpretation of respiratory parameters in these patients.

The recruitable and non-recruitable subphenotypes may aid clinicians in identifying COVID-ARDS patients that likely respond to a recruitment manoeuvre in terms of re-aeration, which is relevant as a recruitment manoeuvre may improve oxygenation in recruitable patients, whereas it might cause damage in non-recruitable patients [9, 27, 28]. However, the effect of recruitment manoeuvres on physiological measures such as compliance, ventilatory ratio and oxygenation has not been addressed in the present study, and it remains speculative whether improvement of these physiological measures would lead to a better outcome. Nonetheless, we found that the subphenotypes can easily be identified using a combination of PaO_2_/FiO_2_ ratio, mechanical power, non-aerated lung mass and normally aerated lung volume at clinical PEEP levels. A practical disadvantage of the described subphenotyping approach is the need for a CT scan, which might be unavailable due to limited resources or problematic transport of hypoxic patients. In those cases, lung ultrasound (LUS) can be an alternative as it is widely available and a recent study showed that it can accurately estimate non-aerated and well-aerated lung tissue [29, 30]. Additionally, the present study established a prediction model without CT-derived parameters that only had a modest loss in accuracy for subphenotype prediction.

## Conclusion

In conclusion, mechanically ventilated COVID-ARDS patients can be divided into subphenotypes that are similar to the recruitable and non-recruitable non-COVID-ARDS subphenotypes. COVID-19 patients with the recruitable subphenotype had a longer duration of mechanical ventilation until successful extubation, while no difference was found in terms of survival. The recruitable and non-recruitable subphenotypes are promising for identification of recruitable patients in future clinical practice as they can be classified with only a few commonly available parameters.

## Supplementary Information

**Additional file 1**: *formulas used*. **Figure S1**: correlation plots. *Stepwise description of discarded variables due to correlation. Missing data.* **Figure S2a**: density plots of imputed variables. **Figure S2b**: strip plots of imputed variables. **Table S1**: main outcomes and transitions between complete case and imputation models. **Figure S3a**: profile plot of all recruitable subphenotypes. **Figure S3b**: profile plot of all non-recruitable subphenotypes. **Figure S4**: alluvial plot of patient flow among models. **Table S2**: changes per lung region. **Figure S5a**: changes in in end-expiratory lung volumes before and after recruitment. **Figure S5b**: changes in lung weight before and after recruitment. **Figure S6a**: volumes in different aeration regions before and after recruitment. **Figure S6b**: weight in different aeration regions before and after recruitment. **Table S3**: LASSO regression results. **Table S4**: GLM results of nested variable models. **Table S5**: AUROCs for variable subsets. **Figure S7**: ROC curves for variable subsets. **Table S6a**: Fine and Gray regression results of subphenotype membership and duration of MV. **Table S6b**: Cox regression results of subphenotype membership and survival. **Figure S8**: Kaplan-Meier plot of survival. **Table S7**. Goodness-of-fit tests. **Figure S9**: Shoenfeld plots for covariates used in survival analysis. **Figure S10**: Cumulative incidence plot using only complete case analyses. **Figure S11**: Kaplan-Meier using only complete cases. **Table S8a**: Fine and Gray regression results of subphenotype membership and duration of MV using complete cases only. **Table S8b**: Cox regression results of subphenotype membership and survival using complete cases only. *References of supplementary materials.*

## Data Availability

The datasets used and/or analysed during the current study are available from the corresponding author on reasonable request.

## Abbreviations

ARDS: Acute respiratory distress syndrome
AUROC: Area under receiver operating characteristics curve
BIC: Bayesian information criterion
CI: Confidence interval
COVID-ARDS: COVID-19-related ARDS
CT: Computed tomography
FiO_2_: Fraction of inspired oxygen
GLM: General linear regression model
HR: Hazard ratio
HU: Hounsfield units
IBW: Ideal body weight
ICU: Intensive care unit
IQR: Interquartile range
LASSO: Least absolute shrinkage and selection operator
LCA: Latent class analysis
LMR-LRT: Lo–Mendell–Rubin adjusted likelihood ratio test
MV: Mechanical ventilation
Non-COVID-ARDS: Non-COVID-19-related ARDS
OR: Odds ratio
P/F ratio: PaO2/FiO2
PaO_2_: Partial pressure of oxygen
PEEP: Positive end expiratory pressure
SD: Standard deviation
SHR: Subdistribution hazard ratio
SMD: Standardized mean difference
TV: Tidal volume
