# Supplementary material for: Latent class analysis of imaging and clinical respiratory parameters from patients with COVID-19-related ARDS identifies recruitment subphenotypes
Source: Crit Care. 2022 Nov 25;26:363. doi: 10.1186/s13054-022-04251-2 (PMC9700924; doi:10.1186/s13054-022-04251-2)
Supplement: Supplementary file 1 — Additional file 1: formulas used. Figure S1: correlation plots. Stepwise description of discarded variables due to correlation. Missing data. Figure S2a: density plots of imputed variables. Figure S2b: strip plots of imputed variables. Table S1: main outcomes and transitions between complete case and imputation models. Figure S3a: profile plot of all recruitable subphenotypes. Figure S3b: profile plot of all non-recruitable subphenotypes. Figure S4: alluvial plot of patient flow among models. Table S2: changes per lung region. Figure S5a: changes in in end-expiratory lung volumes before and after recruitment. Figure S5b: changes in lung weight before and after recruitment. Figure S6a: volumes in different aeration regions before and after recruitment. Figure S6b: weight in different aeration regions before and after recruitment. Table S3: LASSO regression results. Table S4: GLM results of nested variable models. Table S5: AUROCs for variable subsets. Figure S7: ROC curves for variable subsets. Table S6a: Fine and Gray regression results of subphenotype membership and duration of MV. Table S6b: Cox regression results of subphenotype membership and survival. Figure S8: Kaplan-Meier plot of survival. Table S7. Goodness-of-fit tests. Figure S9: Shoenfeld plots for covariates used in survival analysis. Figure S10: Cumulative incidence plot using only complete case analyses. Figure S11: Kaplan-Meier using only complete cases. Table S8a: Fine and Gray regression results of subphenotype membership and duration of MV using complete cases only. Table S8b: Cox regression results of subphenotype membership and survival using complete cases only. References of supplementary materials. [file 13054_2022_4251_MOESM1_ESM.pdf]

Additional file 1: supplementary materials

Contents

1. Formulas used
2. **Figure S1**: correlation plots
3. Stepwise description of discarded variables due to correlation
4. Missing data
5. **Figure S2a**: density plots of imputed variables. Figure S2b: strip plots of imputed variables
6. **Table S1**: main outcomes and transitions between complete case and imputation models
7. **Figure S3a**: profile plot of all recruitable subphenotypes
8. **Figure S3b**: profile plot of all non-recruitable subphenotypes
9. **Figure S4**: alluvial plot of patient flow in complete case and imputation models
10. **Table S2**: changes per lung region
11. **Figure S5a**: changes in in end-expiratory lung volumes before and after recruitment
12. **Figure S5b**: changes in lung weight before and after recruitment
13. **Figure S6a**: volumes in different aeration regions before and after recruitment
14. **Figure S6b**: weight in different aeration regions before and after recruitment
15. **Table S3**: LASSO regression results
16. **Table S4**: GLM results of nested variable models.
17. **Table S5**: AUROCs for variable subsets
    Figure S7: ROC curves for variable subsets
18. **Table S6a**: Fine and Gray regression results of subphenotype membership and duration of MV.
    Table S6b: Cox regression results of subphenotype membership and survival
19. **Figure S8**: Kaplan-Meier plot of survival.
20. **Table S7**. Goodness-of-fit tests.
    Figure S9: Shoenfeld plots for covariates used in survival analysis.
21. **Figure S10**: Cumulative incidence plot using only complete case analyses
22. **Figure S11**: Kaplan-Meier using only complete cases
23. **Table S8a**: Fine and Gray regression results of subphenotype membership and duration of MV using complete cases only.
    Table S8b: Cox regression results of subphenotype membership and survival using complete cases only.
24. References of supplementary materials

Formulas

Ideal Body Weight: Male: $50 + (0.91 \times (Height - 152.4))$ Female: $45.5 + (0.91 \times (Height - 152.4))$

Mechanical Power: $Respiratory\ Rate \times Tidal\ Volume \times \frac{pMax}{1000}$

CO_2 difference: $paCO_2 - etCO_2$

Ventilatory Ratio: $\frac{Respiratory\ Rate \times Tidal\ Volume \times (paCO2 \times 7.50062)}{Weight \times 100 \times 37.5}$

Oxygenation index: $pMap\ \times FiO_2 \times \frac{100}{paO_2}$

Lung weight /volume/aeration: $\sum_{i=1}^{N-1}(d\ \cdot \frac{M_i + M_{i+1}}{2 \cdot t}) + \frac{M_1 + M_N}{2}$

In which N is the number of slices, t is the slice thickness, d is the distance between slices and M_i is the lung mass in the i_{th} slice.

Lung weight: $\left(1 - \frac{mean\ CT\ number}{-\ 1000}\right) \times lung\ volume$

End-expiratory lung volume: $\left(\frac{mean\ CT\ number}{-\ 1000}\right) \times lung\ volume$

Percentage recruitment: $\frac{M_{non-Aerated\ (before\ RM\ )} - M_{non-aerated\ (after\ RM)}}{M_{total\ \ (before\ RM)}} \times 100\%$

In which M is as lung weight in grams and RM indicating recruitment manoeuvre.

Data collection

Clinical respiratory parameters: last values before transport to CT-scan (estimated maximum 30 minutes prior)

Blood gas results: values used in decision making for the recruitment manoeuvre (estimated maximum 3 hours prior)

Laboratory results: morning round (maximum 24 hours prior)

Figure S1. Correlation plots of all variables used in the latent class analysis (left) compared to all available variables before selecting (right). The size of the dots represents the p-value of the correlation (bigger dot equals lower p-value) and the colour represents the correlation coefficient (legend included). Spearman rank-order correlation coefficient was used for calculation. The stars plotted correspond with significance levels; * > 0.05, ** > 0.01, *** > 0.001.

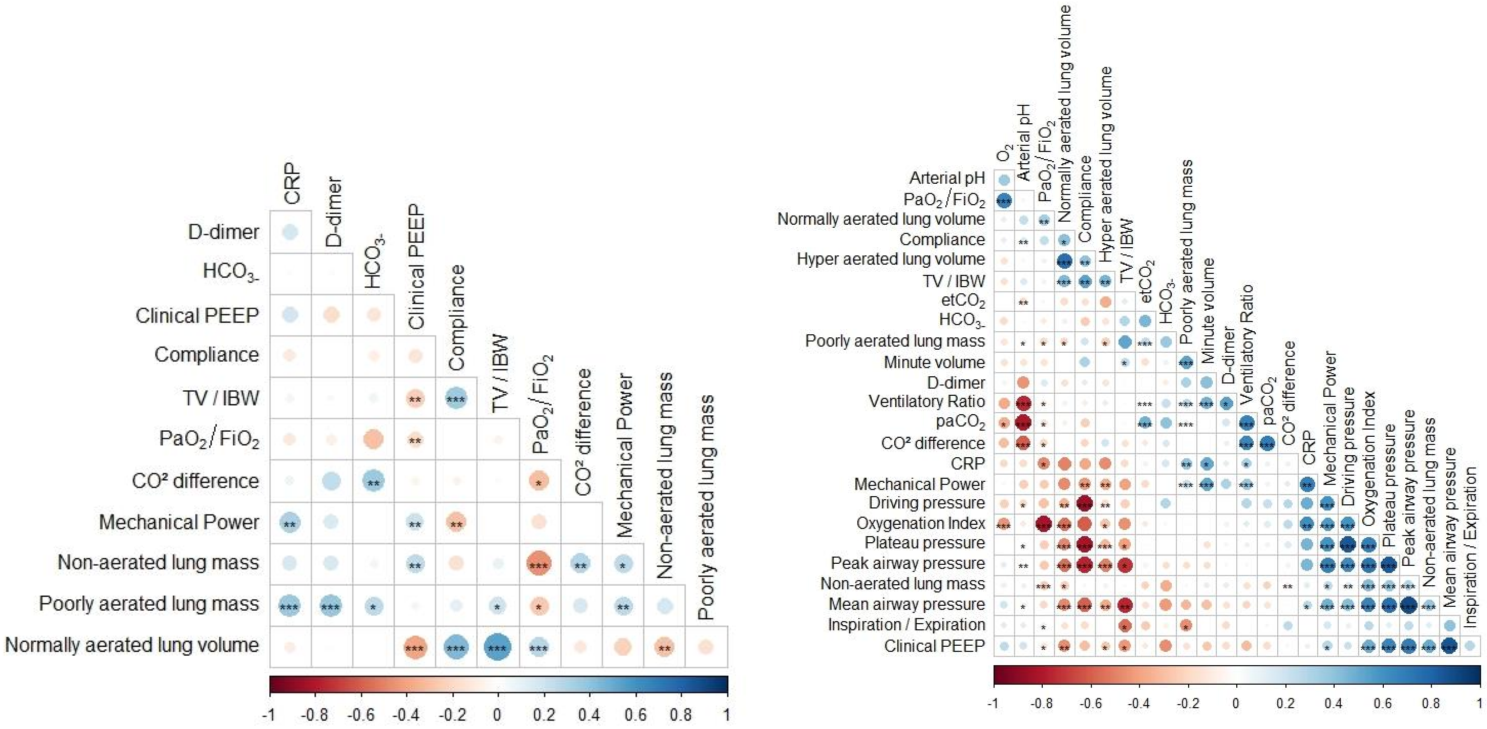

Stepwise selection of variables

At each step, a single strongly correlated variable (correlation coefficient > 0.7, p-value < 0.05) was deleted followed by a recalculation of correlations. In a correlated pair/group, there was a preference for: variables that were clinically more relevant, variables that were used more in comparable literature, more compound variables.

1. Deleted *arterial pH* (correlation with: *CO_2 difference, ventilatory ratio, paCO_2*)
   Preference for other more used and/or more compound variables: *CO_2 difference, ventilatory ratio, paCO_2*
2. Deleted *PaCO_2* (correlation with: *CO_2 difference*)
   Preference for other more used and more compound variable: *CO_2 difference*
3. Deleted *etCO_2* (correlation with: *CO_2 difference, poorly aerated lung mass*)
   etCO2 is used in other variable. Preference for other more used and more compound variable: *CO_2 difference.*
4. Deleted *PaO2* (correlation with: *oxygenation index, PaO_2 / FiO_2*)
   Preference for other more used and more compound variables: *oxygenation index, PaO_2 / FiO_2*
5. Deleted *oxygenation index* (correlation with: *PaO_2 / FiO_2*)
   Preference for other more used variable: *PaO_2 / FiO_2*
6. Deleted *plateau pressure* (correlation with: *compliance, driving pressure, mechanical power*)
   Preference for other more used and more compound variables: *compliance, driving pressure, mechanical power*
7. Deleted *driving pressure* (correlation with: *compliance, mechanical power*)
   Preference for other more used and more compound variables: *compliance, mechanical power*
8. Deleted *peak airway pressure* (correlation with: *mechanical power, compliance, mean airway pressure*)
   Preference for other more used and more compound variables: *mechanical power, compliance, mean airway pressure*
9. Deleted *mean airway pressure* (correlation with: *mechanical power, compliance*)
   Preference for other more used and more compound variables: *mechanical power, compliance*
10. Deleted *minute volume* (correlation with: *poorly aerated lung volume, mechanical power, ventilatory ratio*)
    Preference for other more used and more compound variables: *mechanical power, ventilatory ratio*
11. Deleted *ventilatory ratio* (correlation with: *mechanical power, CO_2 difference*)
    Preference for other more used and more compound variables: *mechanical power, CO_2 difference*
12. Deleted *I/E ratio*
    Homogenous variable, not well distributed, not used in comparable literature
13. Deleted *hyper aerated lung volume* (correlation with *normally aerated lung volume*)
    Although both important, hyper aerated lung volume is less reliable.

Missing data

As LCA requires complete datasets, missing data was imputed by fully conditional specification with predictive mean matching under the missing at random assumption[1,2]. Imputation was limited to the variables used in the LCA, other variables and outcomes were not imputed. Prediction of imputed variables was based only on variables used in the LCA. Five imputation models with a maximum of 50 iterations each were created. The quality of the imputation models was visually assessed using stripplots and density plots for each imputated variable[2,3]. The effects of imputation on latent class assignment were tested by calculating class transitions between complete case and imputation models. Outcomes and class characteristics were compared among complete case and imputation models.

Figure S2a. Density plots of variables in complete case dataset compared to imputated datasets. Complete case is plotted in black, colored lines are different imputation sets. Only imputated variables are included.

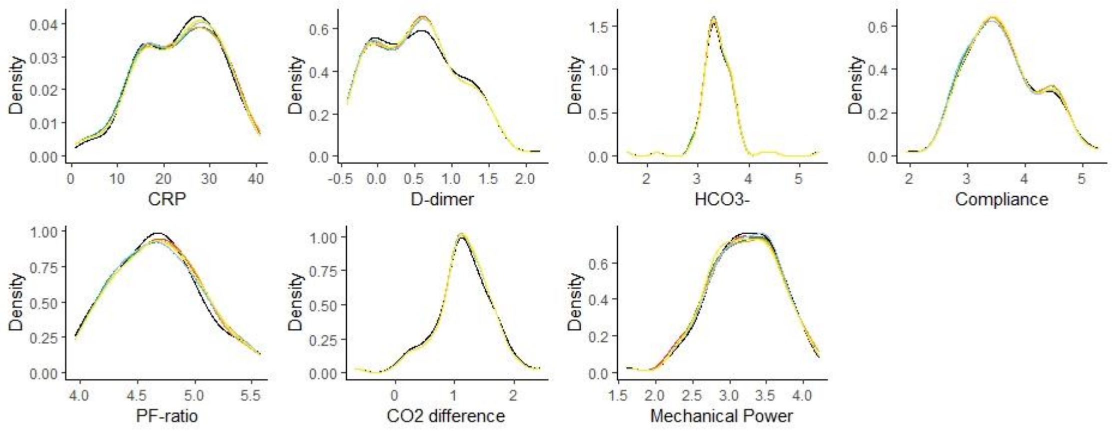

Figure S2b. Stripplots of imputated variables only. Complete case data is displayed with grey dots, imputated datapoints are colored.

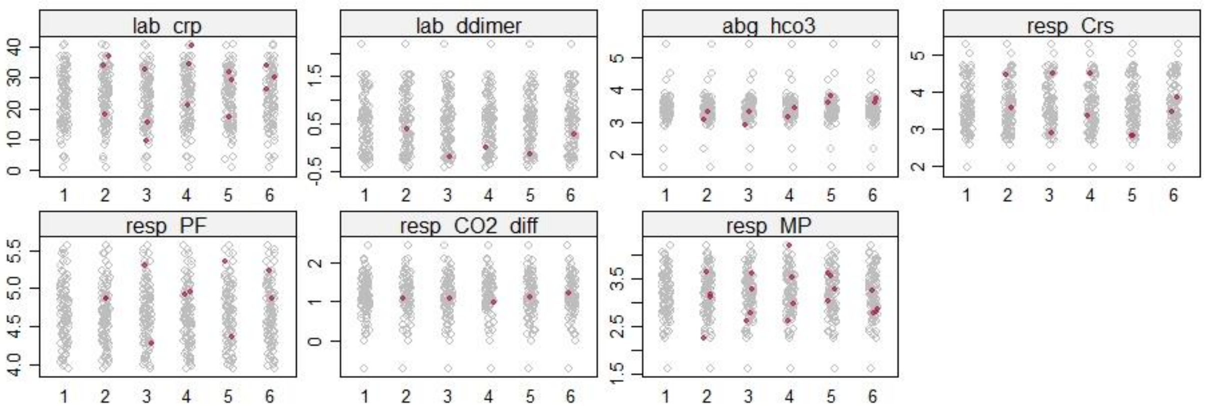

Table S1: Main outcomes and transitions between complete case and imputation models. Data is displayed according to distribution.

	Complete case			Model 1			Model 2			Model 3			Model 4			Model 5		
**Class**	1	2		1	2		1	2		1	2		1	2		1	2	
**N**	52	38		62	37		61	38		60	39		63	36		60	39	
			p			p			p			p			p			p
**Recruitability %**	9.22 [4.34, 12.62]	12.02 [5.30, 17.70]	0.102	8.95 [3.68, 14.25]	12.56 [6.72, 18.17]	0.024	9.11 [3.76, 14.30]	12.02 [6.17, 18.10]	0.059	9.22 [3.74, 14.33]	11.48 [6.35, 18.04]	0.072	9.11 [4.15, 13.77]	14.61 [6.54, 19.19]	0.021	8.95 [3.74, 14.14]	12.56 [6.35, 18.48]	0.029
**Duration of mechanical ventilation**	13.50 [7.00, 22.75]	16.00 [12.00, 27.00]	0.149	15.00 [7.50, 25.50]	16.00 [12.00, 27.00]	0.189	15.50 [7.25, 25.75]	16.00 [11.50, 27.00]	0.278	15.50 [7.25, 25.75]	16.00 [11.50, 27.00]	0.278	17.00 [7.50, 26.50]	15.00 [10.25, 25.25]	0.631	16.00 [7.00, 26.00]	15.50 [11.00, 27.00]	0.373
**Duration of ICU stay**	18.50 [9.75, 29.25]	18.00 [13.00, 28.00]	0.263	18.00 [10.00, 30.50]	17.50 [13.00, 32.50]	0.266	19.00 [10.00, 30.75]	17.00 [12.50, 31.00]	0.411	19.00 [10.00, 30.75]	17.00 [12.50, 31.00]	0.411	20.00 [10.00, 31.00]	17.00 [12.25, 27.50]	0.743	20.00 [10.00, 31.00]	17.00 [12.00, 29.50]	0.551
**Extubation succes (%)**	24 (54.5)	13 (35.1)	0.128	27 (52.9)	12 (35.3)	0.168	27 (54.0)	12 (34.3)	0.115	27 (54.0)	12 (34.3)	0.115	26 (51.0)	13 (38.2)	0.351	26 (53.1)	13 (36.1)	0.184
**ICU mortality (%)**	19 (43.2)	22 (59.5)	0.216	23 (45.1)	20 (58.8)	0.308	22 (44.0)	21 (60.0)	0.218	22 (44.0)	21 (60.0)	0.218	24 (47.1)	19 (55.9)	0.565	22 (44.9)	21 (58.3)	0.315
**Transitions between classes, compared to model 1**	2	5		-	-		1	0		2	0		3	4		2	0	

Figure S3a: profile plot of all recruitable subphenotypes in complete case (CC) and imputation models (I1 – I5). Variables used in the LCA are displayed on the X-axis and their corresponding standardized mean difference (SMD) on the Y-axis.

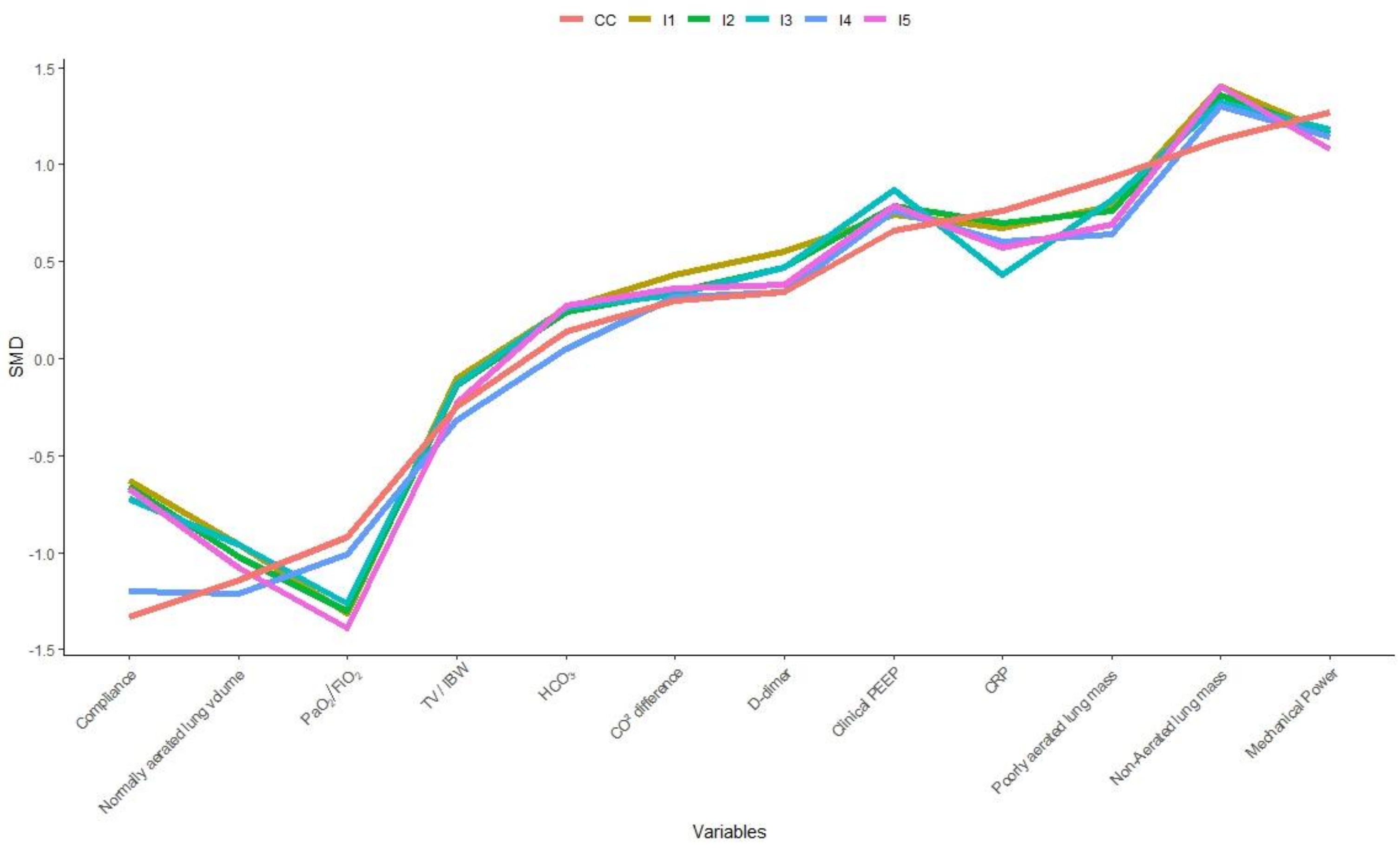

Figure S3b: Profile plot of all non-recruitable subphenotypes in complete case (CC) and imputation models (I1 – I5). Variables used in the LCA are displayed on the X-axis and their corresponding standardized mean difference (SMD) on the Y-axis.

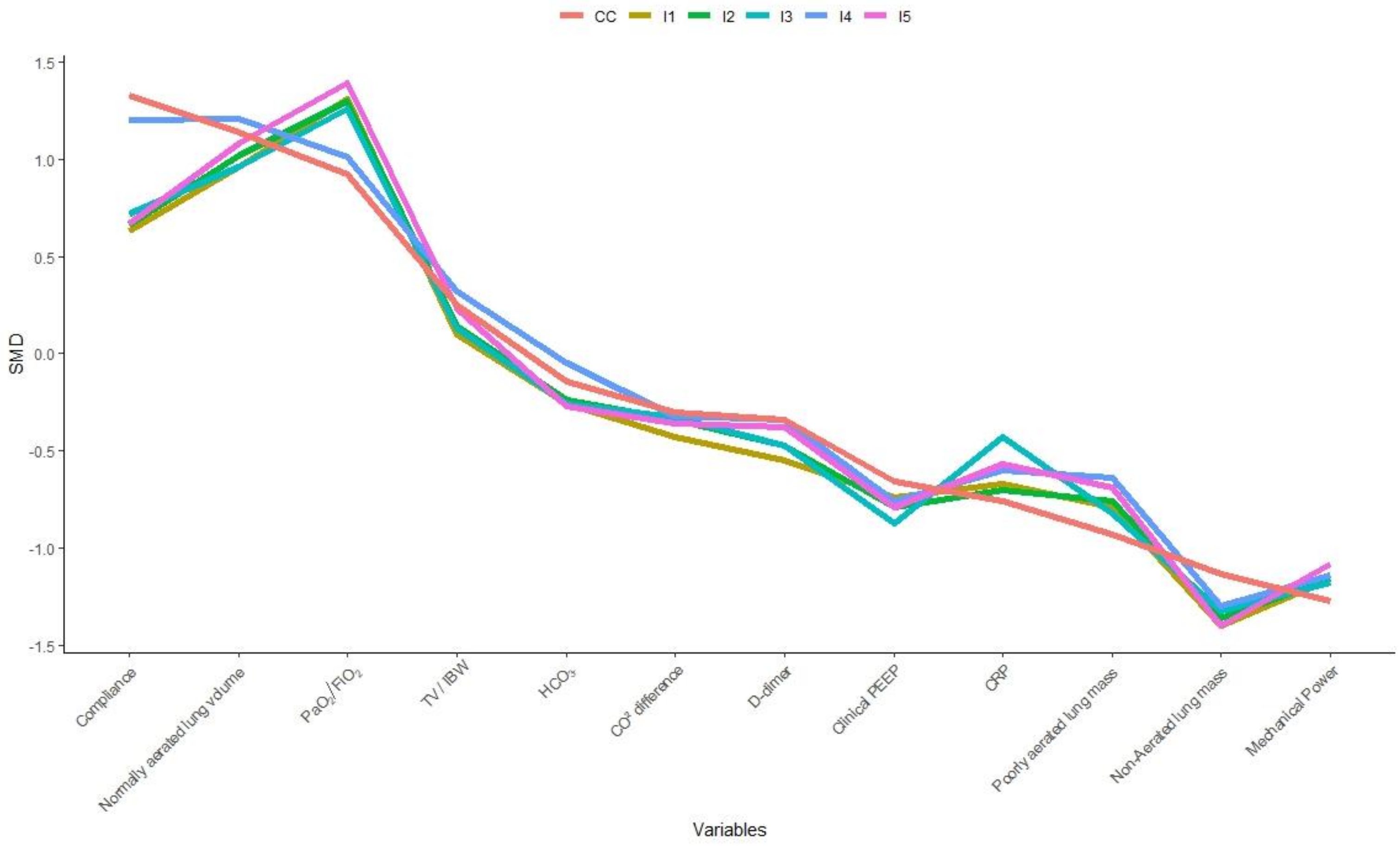

Figure S4. Alluvial plot of complete case (left) and imputation models (subsequent 5). Patient flow is coloured according to class membership in complete case analysis.

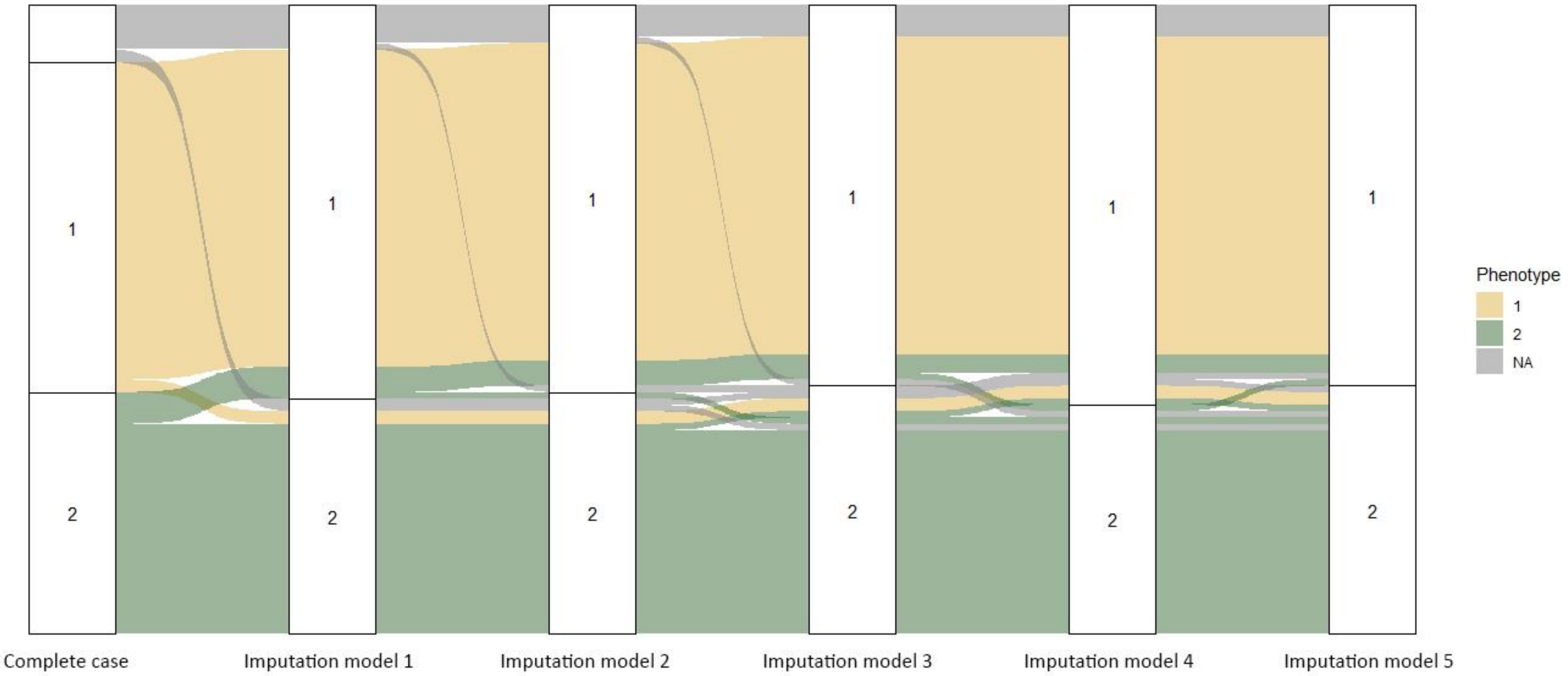

Table S2. Changes per lung region. All values displayed according to distribution.

	Subphenotype 1 (n=62)<br>Before	"Non-recruitable"<br>After	p-value	Subphenotype 2 (n=37)<br>Before	"Recruitable"<br>After	p-value	Between subphenotypes p-value
**PEEP**	10.00 [10.00, 10.00]	20.00 [20.00, 20.00]	<0.001	10.00 [10.00, 10.00]	20.00 [20.00, 20.00]	<0.001	
**Total lung volume**	2843.10 [2175.11, 3736.62]	3667.68 [2687.67, 4347.86]	0.002	2597.61 [2258.73, 3220.69]	3272.79 [2653.50, 3917.91]	0.004	
**Total lung mass**	1326.56 [1030.22, 1619.78]	1360.51 [1086.23, 1614.42]	0.640	1613.68 [1429.67, 1884.76]	1724.21 [1511.19, 1927.12]	0.240	
**Lung volume non-aerated**	259.36 [181.03, 406.27]	140.72 [85.60, 227.96]	<0.001	576.96 [392.14, 735.77]	341.78 [234.89, 466.79]	<0.001	
**Lung mass normal**	439.04 [322.85, 612.25]	674.27 [477.25, 780.09]	<0.001	260.05 [161.19, 420.02]	444.24 [301.73, 693.93]	0.001	
**Percentage volume non-aerated**	11.29 [6.09, 15.17]	3.65 [2.46, 6.53]	<0.001	22.96 [14.11, 30.96]	9.05 [6.96, 13.91]	<0.001	
Change %	-4.09 [-7.78, -1.65]			-8.12 [-12.31, -3.75]			0.002
**Percentage volume poorly aerated**	26.40 [19.42, 35.25]	20.63 [13.47, 28.64]	<0.001	35.19 [30.06, 51.63]	38.04 [28.95, 51.69]	0.698	
Change %	-1.85 [-5.82, 0.62]			6.47 [1.01, 12.50]			<0.001
**Percentage volume normally aerated**	56.01 [45.89, 65.36]	66.71 [58.81, 76.27]	<0.001	35.42 [19.51, 45.36]	46.88 [36.21, 58.85]	<0.001	
Change %	24.10 [14.80, 43.24]			22.83 [12.35, 31.00]			0.360
**Percentage volume hyper aerated**	1.31 [0.20, 3.91]	2.97 [0.69, 6.81]	<0.001	0.16 [0.03, 0.80]	0.62 [0.20, 1.64]	<0.001	
Change %	1.09 [0.37, 3.81]			0.27 [0.15, 1.32]			0.009
**Percentage mass non-aerated**	22.21 [14.69, 30.24]	10.01 [6.46, 15.30]	<0.001	35.81 [24.88, 43.74]	17.53 [13.94, 25.33]	<0.001	
Change %	-8.95 [-14.25, -3.68]			-12.56 [-18.17, -6.72]			0.024
**Percentage mass poorly aerated**	38.56 [31.40, 45.99]	33.37 [26.32, 41.86]	<0.001	43.47 [30.83, 54.68]	47.43 [36.47, 57.94]	0.017	
Change %	-3.38 [-8.60, 0.90]			6.09 [0.97, 10.75]			<0.001
**Percentage mass normally aerated**	33.17 [27.19, 44.76]	50.28 [39.79, 60.81]	<0.001	15.01 [11.38, 24.07]	27.22 [18.88, 39.03]	<0.001	
Change %	13.53 [7.44, 22.81]			11.56 [6.45, 16.93]			0.231
**Percentage mass hyper aerated**	0.21 [0.03, 0.76]	0.59 [0.15, 1.62]	<0.001	0.02 [0.00, 0.12]	0.06 [0.02, 0.28]	<0.001	
Change %	0.18 [0.06, 0.66]			0.03 [0.01, 0.22]			0.002

Figure S5a. Changes in end-expiratory lung volumes before and after the recruitment manoeuvre. Data is stratified per lung aeration category and per subphenotype. Lung regions were classified according to Hounsfield Units (HU) with normally aerated ranging from −900 to −501 HU, poorly aerated from −500 to −101 HU, non-aerated from −100 to 100 HU and hyper-inflated from -1000 to -901 HU.

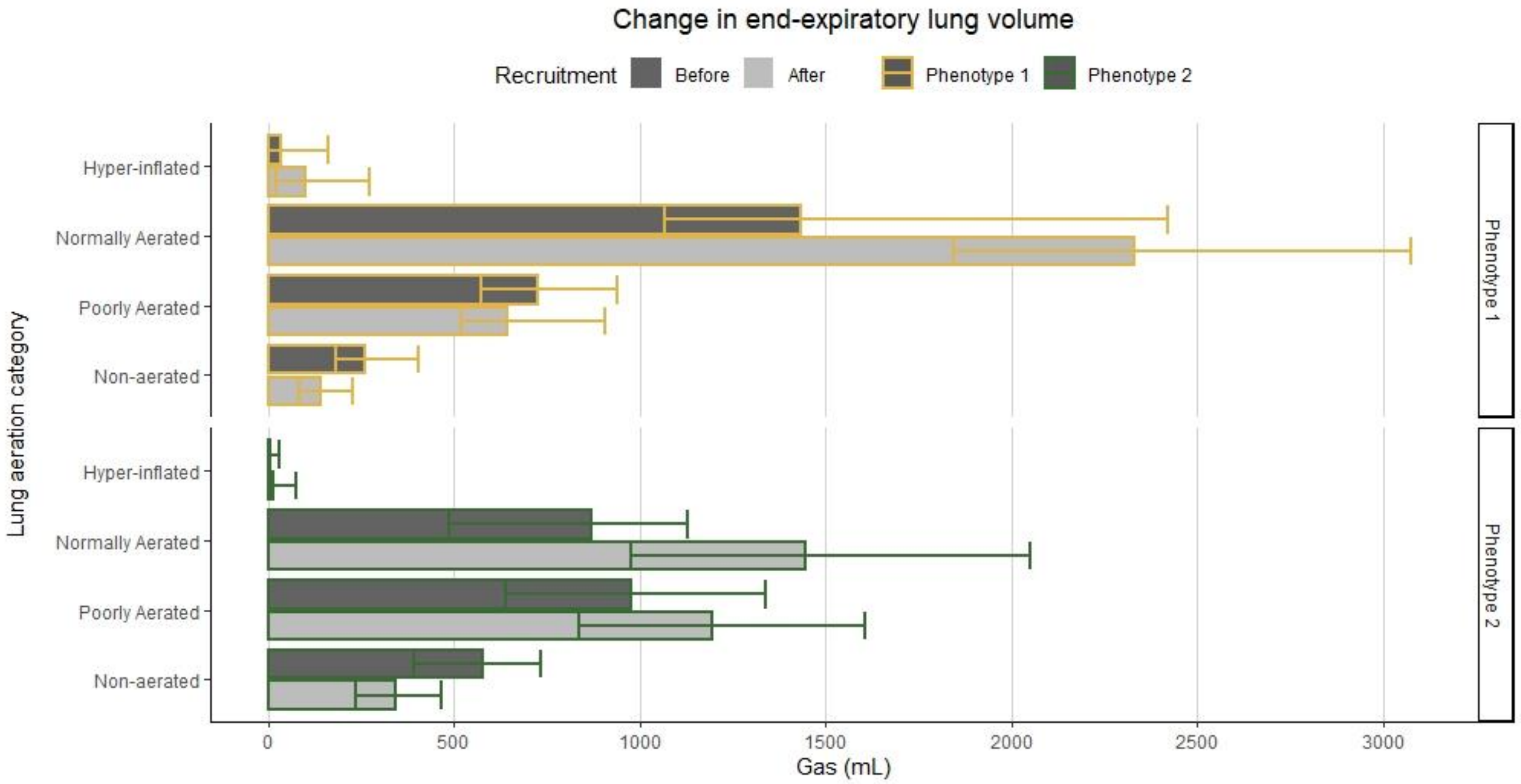

Figure S5b. Changes in lung weight before and after the recruitment manoeuvre. Data is stratified per lung aeration category and per subphenotype. Lung regions were classified according to Hounsfield Units (HU) with normally aerated ranging from −900 to −501 HU, poorly aerated from −500 to −101 HU, non-aerated from −100 to 100 HU and hyper-inflated from -1000 to -901 HU.

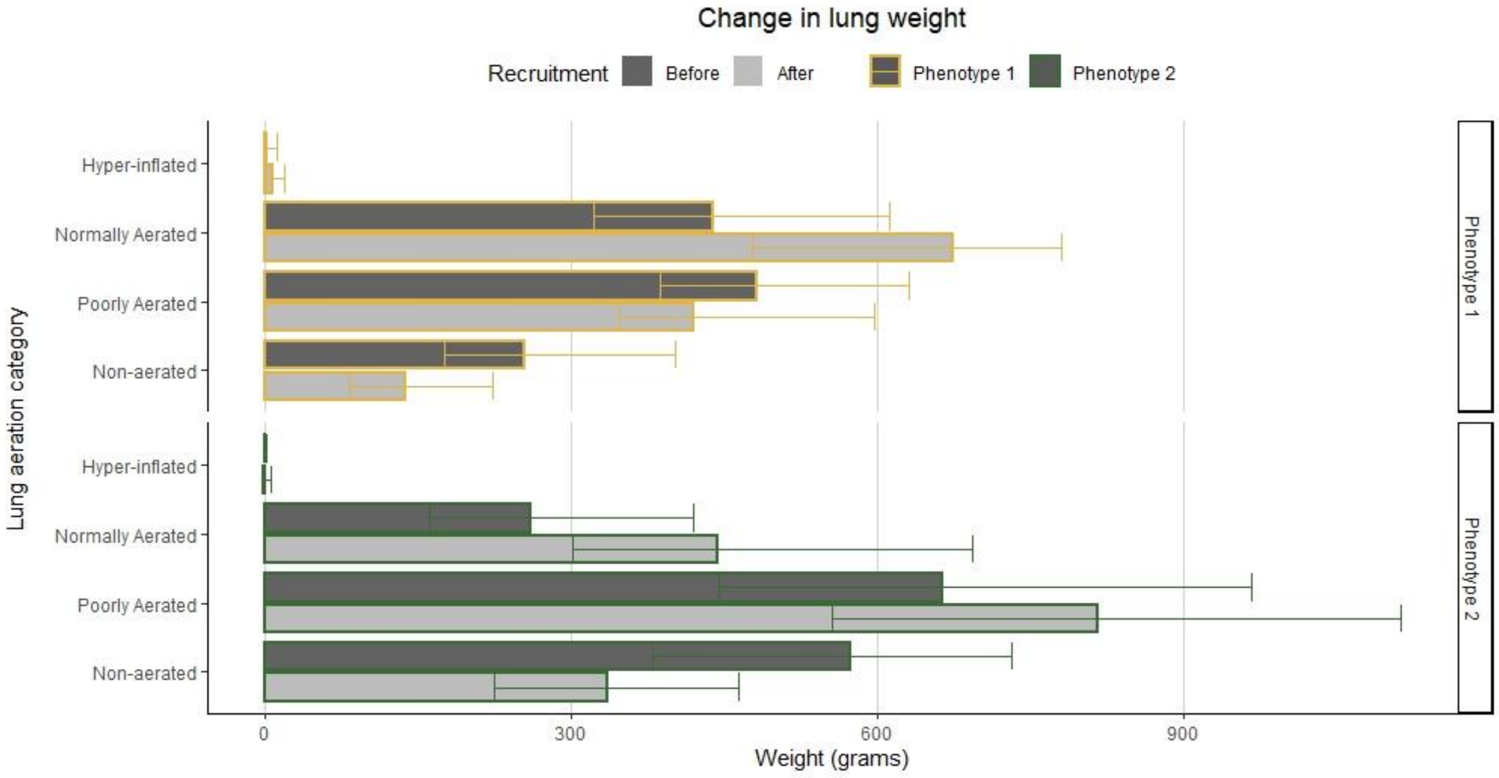

Figure S6a. End-expiratory lung volume (in mL) of different aeration regions displayed before and after the recruitment manoeuvre. Corresponding patient data points are connected by a line. Data is stratified and coloured by subphenotype. The p-values before and after recruitment (bottom p-values) compare relative amounts (mL / total mL) by the recruitment manoeuvre and are derived by Wilcoxon signed-rank tests. The p-values between subphenotypes (upper p-values) compare the changes in relative amounts by the recruitment manoeuvre (change in mL / total mL) between subphenotypes and are derived by Mann-Whitney U tests (table S2).

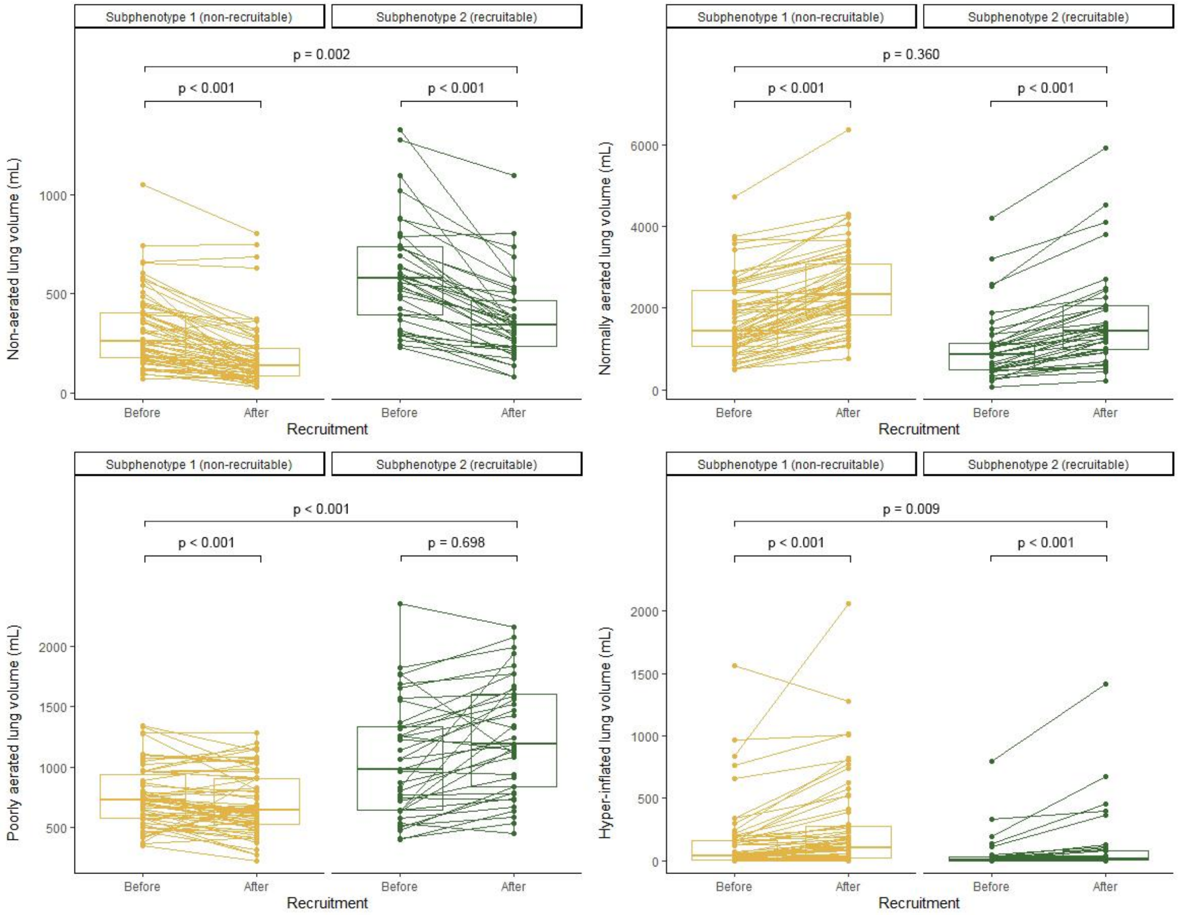

Figure S6b. Mass (in grams) of different aeration regions displayed before and after the recruitment manoeuvre. Corresponding patient data points are connected by a line. Data is stratified and coloured by subphenotype. The p-values before and after recruitment (bottom p-values) compare relative amounts (grams / total grams) by the recruitment manoeuvre and are derived by Wilcoxon signed-rank tests. The p-values between subphenotypes (upper p-values) compare the changes in relative amounts by the recruitment manoeuvre (change in grams / total grams) between subphenotypes and are derived by Mann-Whitney U tests (table S2).

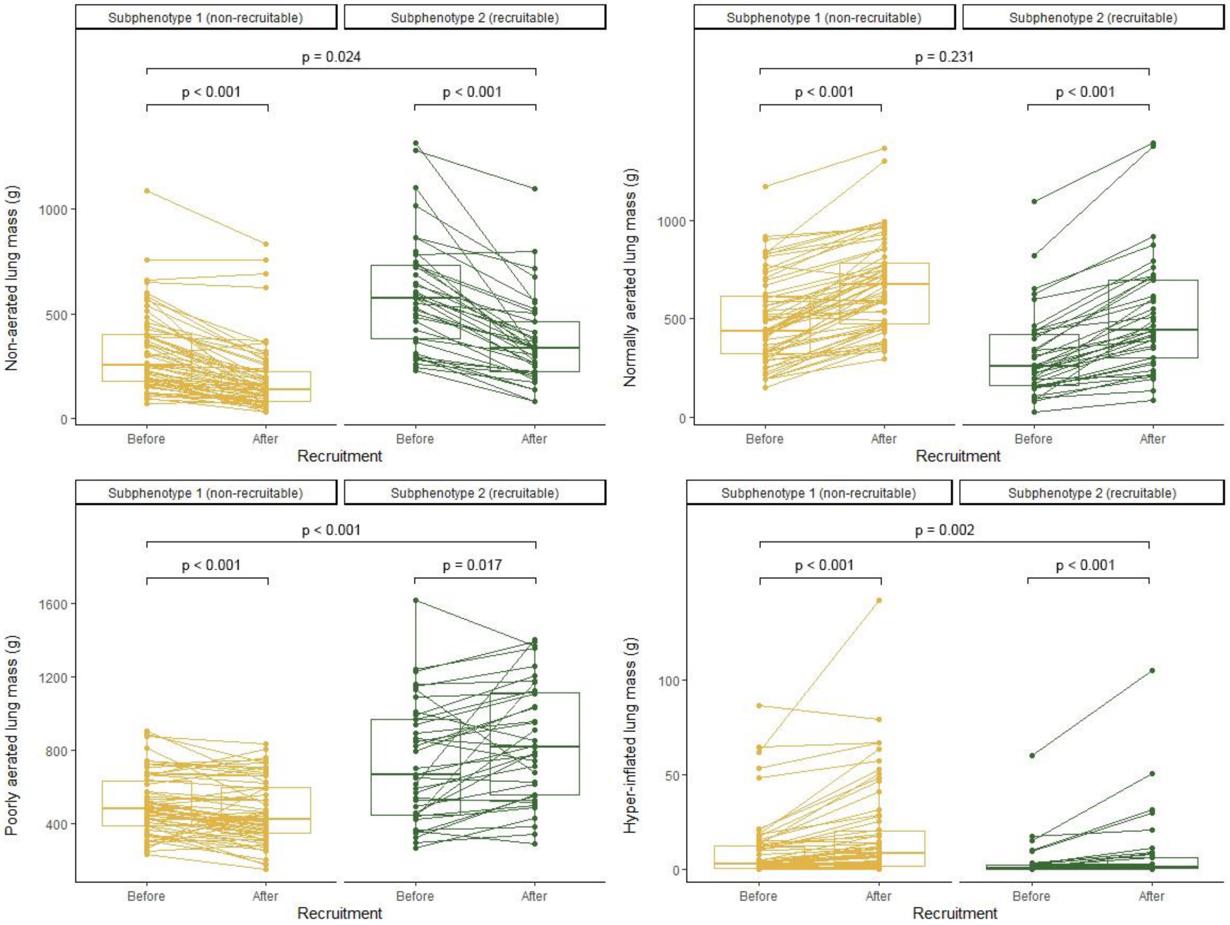

Subphenotype identification using variable subsets and standard ICU severity scores.

Table S3. LASSO coefficient estimates, with the lambda parameter tuned in order to arrive at 4 variables.

	**S0**
Prediction model considering all variables	
Intercept	0.5655465
CRP	-
D-Dimer	-
HCO3-	-
Clinical PEEP	-
Compliance	-
TV / IBW	-
PaO_2 / FiO_2	0.2533840
CO_2 difference	-
Mechanical Power	-0.2732269
Non-aerated lung mass	-0.3070397
Poorly aerated lung mass	-
Normally aerated lung volume	0.0665896
Prediction model without CT-derived parameters	
Intercept	0.63997659
CRP	-0.02684768
D-Dimer	-
HCO3-	-
Clinical PEEP	-
Compliance	0.10852415
TV / IBW	-
PaO2 / FiO2	0.71079442
CO2 difference	-
Mechanical Power	-0.60568786
Intercept	0.63997659
CRP	-0.02684768
D-Dimer	-

Table S4. GLM results (validated with 5-fold cross validation) of selected variable models in terms of subphenotype prediction.

	**Estimate**	**St. Error**	**Z-value**	**P-value**
**Prediction model (n=4) considering all variables**				
Intercept	1.3157	0.4071	3.232	0.00123*
Non-aerated lung mass	-1.4833	0.5438	-2.728	0.00638*
Mechanical power	-1.7986	0.4963	-3.240	0.00029*
PaO_2 / FiO_2	1.1801	0.4312	2.737	0.00620*
Normally aerated lung volume	1.0497	0.3922	2.677	0.00744*
**Prediction model (n=4) considering all variables excluding CT-derived parameters**				
Intercept	1.1848	0.3675	3.224	0.00126*
Mechanical power	-1.2666	0.4065	-3.116	0.00183*
PaO_2 / FiO_2	1.8136	0.4388	4.133	< 0.0001*
Compliance	0.8463	0.3638	2.326	0.02001*
CRP	-0.5871	0.3509	-1.673	0.09427
**PaO_2 / FiO_2**				
Intercept	0.7453	0.2595	2.872	0.00408
PaO_2 / FiO_2	1.4467	0.3219	4.494	< 0.00001
**Apache II**				
Intercept	0.57260	0.62810	0.912	0.3620
Apache II	-0.06558	0.03641	-1.801	0.0716
**SOFA**				
Intercept	-0.96399	0.57954	-1.663	0.0962
SOFA	0.06650	0.07576	0.878	0.3801

Table S5. Areas under the receiver operating curves (AUROCs) for the variable subsets and standard severity scores, in terms of subphenotype prediction. 95% confidence intervals are derived by means of bootstrapping the AUROCs.

	**AUROC (95% CI)**
**Prediction model (n=4) considering all variables**	
Non-aerated lung mass, mechanical power, PaO_2/FiO_2, normally aerated lung volume	0.93 (0.88-0.98)
**Prediction model (n=3) considering all variables excluding CT-derived parameters**	
Mechanical power, PaO_2/FiO_2, compliance	0.87 (0.79-0.91)
**Standard severity score**	
PaO_2/FiO_2	0.79 (0.56-0.82)
Apache II	0.62 (0.48-0.72)
SOFA	0.51 (0.34-0.58)

Figure S7. Receiver operating characteristic (ROC) curves for variable subsets in terms of phenotype prediction. AUROC are listed in table S5.

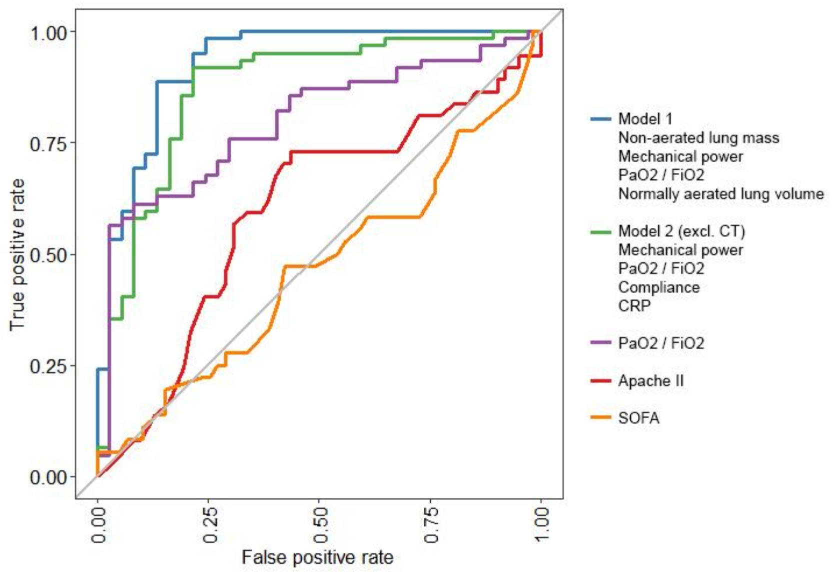

Table S6b. Fine and Grey regression results of subphenotype membership and duration of mechanical ventilation

	SHR (95% CI)	P- value
**Univariate analysis of subphenotype and Mechanical ventilation duration**		
Subphenotype 2 (recruitable)	0.56 (0.23 – 1.07)	p = 0.077
**Multivariate analysis of subphenotype + Confounders, and mechanical ventilation duration**		
Subphenotype 2 (recruitable)	0.46 (0.23 - 0.91)	p = 0.026
Age	0.97 (0.94 – 1.00)	p = 0.073
Gender (male)	0.74 (0.34 - 1.59)	p = 0.430
Apache II score	0.97 (0.92 - 1.03)	p = 0.320

Table S6b. Cox regression results of subphenotype membership and survival

	HR (95% CI)	P- value
**Univariate analysis of subphenotype, and mortality**		
Subphenotype 1 (non-recruitable)	1.08 (0.58 - 1.98)	p = 0.814
**Multivariate analysis of subphenotype + confounders, and mortality**		
Subphenotype 1 (non-recruitable)	1.18 (0.6 - 2.34)	p = 0.629
Age	1.02 (0.97 - 1.07)	p = 0.461
Gender (male)	0.91 (0.43 - 1.92)	p = 0.799
Apache II score	1.01 (0.95 - 1.07)	p = 0.816

Figure S8. Kaplan-Meier plot of survival.

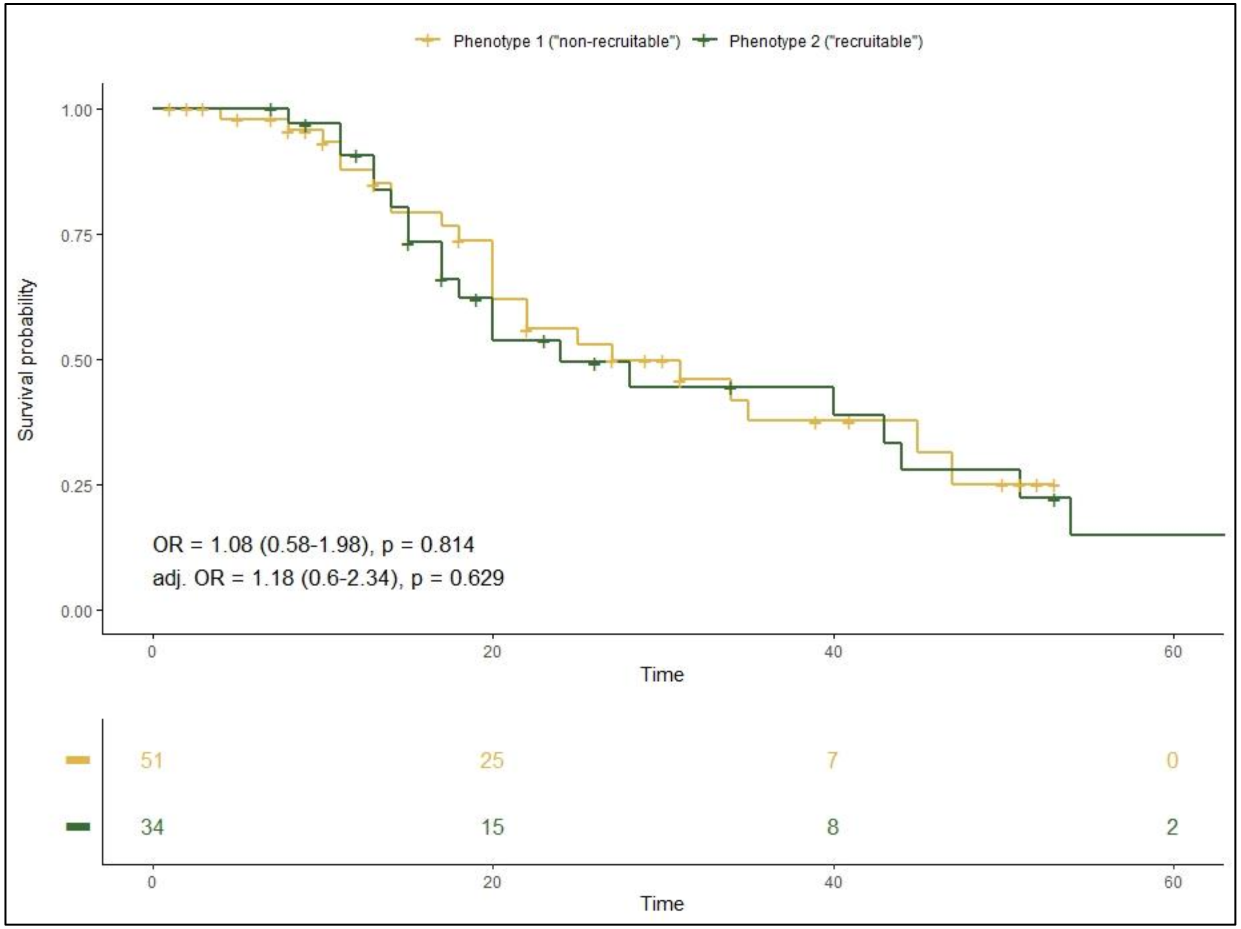

Table S7. Goodness-of-fit tests; tests correlation between Schoenfeld residuals and time. A p-value above 0.05 indicates a high likelihood that hazards are proportional.

	Chi-square	df	p
**Subphenotype**	0.0407	1	0.840
**Age**	0.2439	1	0.621
**Gender**	4.8818	1	0.027
**Apache 2**	0.9898	1	0.320
**Global**	8.5271	4	0.074

Figure S9. Shoenfeld residuals plots for covariates used in the survival analysis. For each moment in time, residuals are displayed by the black line with the 95% CI in dotted lines around it. A good proportional model is centered (with its 95% CI) around zero most of the time.

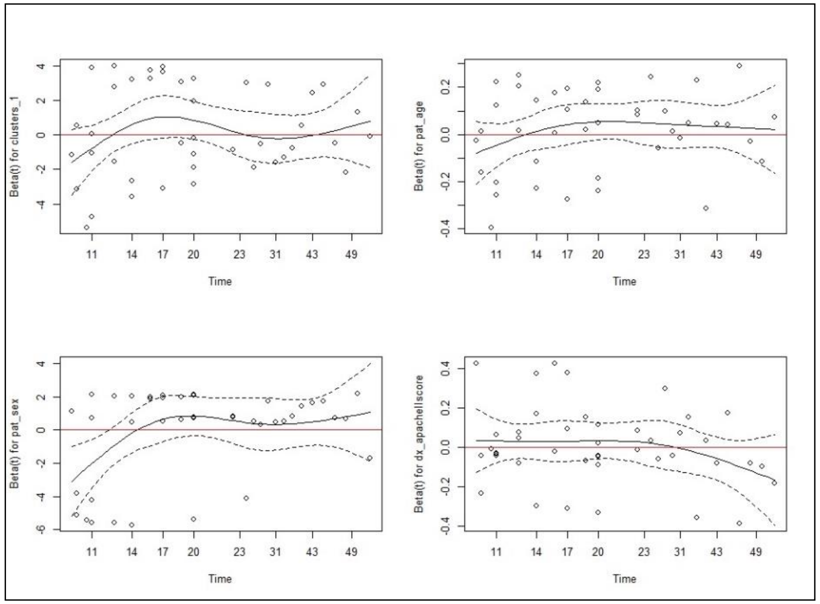

Figure S10. Cumulative incidence plot using only complete cases of time-to-event analysis (time = MV duration, event = successful extubation) in the presence of survival.

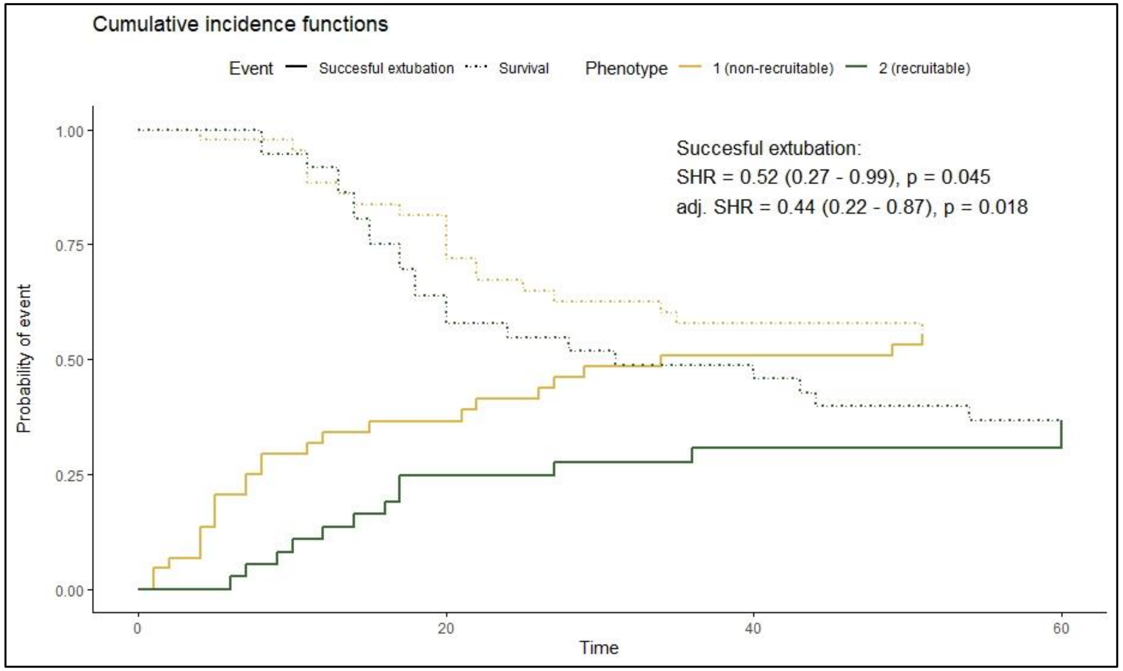

Figure S11. Kaplan-Meier plot using only complete cases of survival analysis.

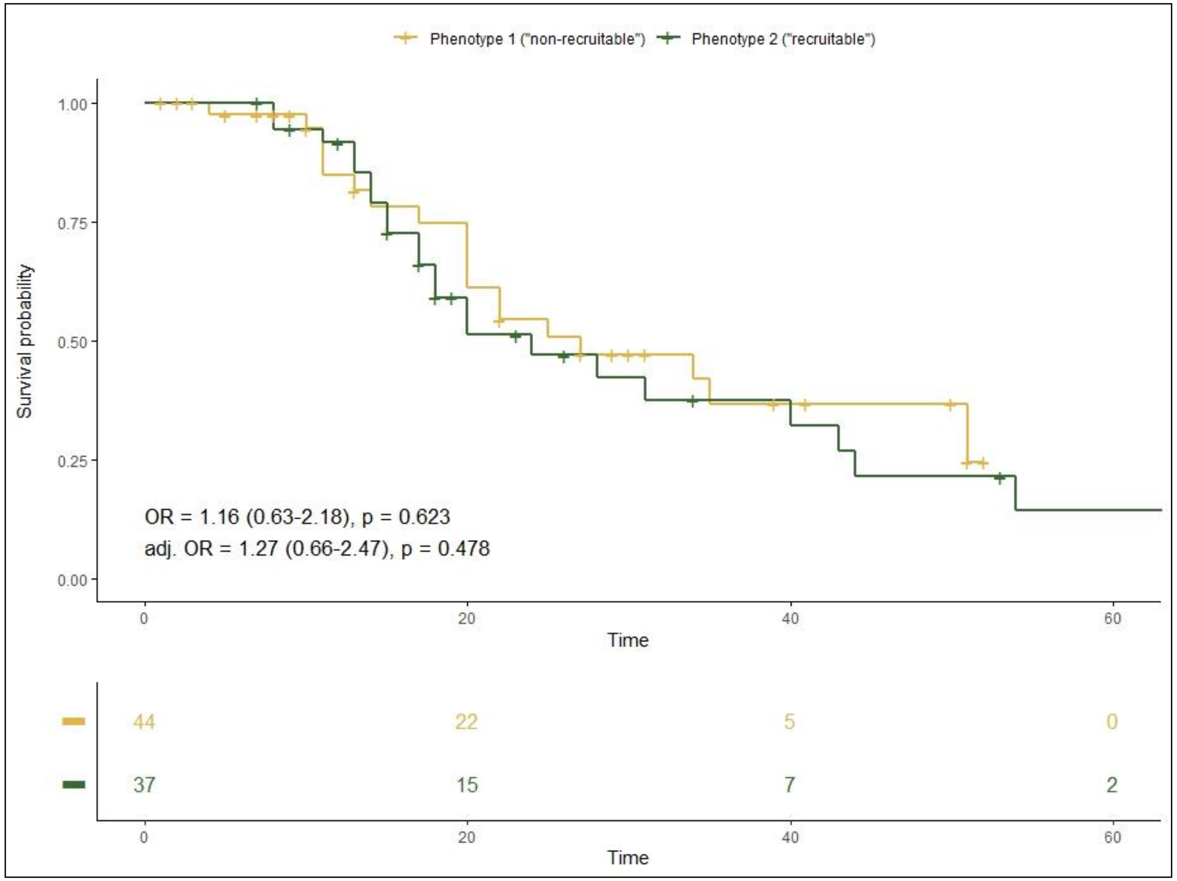

Table S8a. Fine and Gray regression results of subphenotype membership and outcomes in complete case patients only

	HR (95% CI)	P- value
**Univariate analysis of subphenotype and Mechanical ventilation duration**		
Subphenotype 1 (non-recruitable)	0.52 (0.27 - 0.99)	p = 0.045
**Multivariate analysis of subphenotype + Confounders, and mechanical ventilation duration**		
Subphenotype 1 (non-recruitable)	0.44 (0.22 - 0.87)	p = 0.018
Age	0.98 (0.95 - 1.01)	p = 0.210
Gender (male)	0.69 (0.32 - 1.52)	p = 0.360
Apache II score	0.96 (0.90 - 1.02)	p = 0.230

Table S8b. Cox regression results of subphenotype membership and outcomes in complete case patients only

	HR (95% CI)	P- value
**Univariate analysis of subphenotype, and mortality**		
Subphenotype 1 (non-recruitable)	1.16 (0.63 - 2.18)	p = 0.623
**Multivariate analysis of subphenotype + confounders, and mortality**		
Subphenotype 1 (non-recruitable)	1.27 (0.66 - 2.47)	p = 0.478
Age	1.03 (0.98 - 1.08)	p = 0.309
Gender (male)	0.97 (0.46 - 2.05)	p = 0.939
Apache II score	1 (0.94 - 1.06)	p = 0.977

References (supplementary materials)

1. Sinha, P., Calfee, C. S., & Delucchi, K. L. (2021). Practitioner's Guide to Latent Class Analysis: Methodological Considerations and Common Pitfalls. *Crit Care Med*, *49*(1), e63-e79. https://doi.org/10.1097/ccm.0000000000004710
2. van Buuren, S. (2007). Multiple imputation of discrete and continuous data by fully conditional specification. *Statistical Methods in Medical Research*, *16*(3), 219-242. https://doi.org/10.1177/0962280206074463
3. Abayomi, K., Gelman, A., & Levy, M. (2005). Diagnostics for multivariate imputations. *Applied Statistics*, *57*, 273-291.
